# Pan-Genomics of *Escherichia albertii* for Antibiotic Resistance Profiling in Different Genome Fractions and Natural Product Mediated Intervention: In Silico Approach

**DOI:** 10.3390/life13020541

**Published:** 2023-02-15

**Authors:** Khurshid Jalal, Kanwal Khan, Ajmal Hayat, Sulaiman Mohammed Alnasser, Alotaibi Meshal, Zarrin Basharat

**Affiliations:** 1 H.E.J. Research Institute of Chemistry, International Center for Chemical and Biological Sciences, University of Karachi, Karachi 75270, Pakistan; 2 Dr. Panjwani Center for Molecular Medicine and Drug Research, International Center for Chemical and Biological Sciences, University of Karachi, Karachi 75270, Pakistan; 3 Department of Pharmacy, Abdul Wali Khan University, Mardan 23200, Pakistan; 4 Department of Pharmacology and Toxicology, Unaizah College of Pharmacy, Qassim University, Buraydah 52571, Saudi Arabia; 5 Department of Pharmacy Practice, College of Pharmacy, University of Hafr Albatin, Hafar Al Batin 39524, Saudi Arabia; 6 Jamil-ur-Rahman Center for Genome Research, Dr. Panjwani Center for Molecular Medicine and Drug Research, International Center for Chemical and Biological Sciences, University of Karachi, Karachi 75270, Pakistan

**Keywords:** emerging pathogens, *Escherichia albertii*, phytochemicals, ZipA, traditional Chinese medicine, Ayurvedic medicine, pharmcokinetics

## Abstract

*Escherichia albertii* is an emerging, enteric pathogen of significance. It was first isolated in 2003 from a pediatric diarrheal sample from Bangladesh. In this study, a comprehensive in silico strategy was followed to first list out antibiotic-resistant genes from core, accessory and unique genome fractions of 95 available genomes of *E. albertii*. Then, 56 drug targets were identified from the core essential genome. Finally, ZipA, an essential cell division protein that stabilizes the FtsZ protofilaments by cross-linking them and serves as a cytoplasmic membrane anchor for the Z ring, was selected for further downstream processing. It was computationally modeled using a threading approach, followed by virtual screening of two phytochemical libraries, Ayurvedic (n = 2103 compounds) and Traditional Chinese Medicine (n = 36,043 compounds). ADMET profiling, followed by PBPK modeling in the central body compartment, in a population of 250 non-diseased, 250 cirrhotic and 250 renally impaired people was attempted. ZINC85624912 from Chinese medicinal library showed the highest bioavailability and plasma retention. This is the first attempt to simulate the fate of natural products in the body through PBPK. Dynamics simulation of 20 ns for the top three compounds from both libraries was also performed to validate the stability of the compounds. The obtained information from the current study could aid wet-lab scientists to work on the scaffold of screened drug-like compounds from natural resources and could be useful in our quest for therapy against antibiotic-resistant *E. albertii*.

## 1. Introduction

*Escherichia albertii* is a mucocutaneous, non-motile, monophyletic bacterium. It is an etiologic agent of foodborne illness and diarrhea [1,2,3]. Mainly responsible for the implication of bacteremia [4], it was initially isolated from a Bengali infant suffering from diarrhea. It was primarily demarcated as an eae-positive *Hafnia alvei* [5]. Later characterization showed that it belonged to the genus *Escherichia* and consisted of virulence genes (eae and cdt) and a type III secretion system (T3SS). It was renamed *E. albertii* [6] and considered an emerging pathogen since the number of cases manifesting the pathogen-associated disease has risen in several countries [3,7,8]. This bacterium exhibits antimicrobial resistance, with a large fraction (62.7%) of known strains presenting tetracycline resistance [1,9,10]. Resistance to cephalothin, kanamycin [11], monobactam, chloramphenicol, cephalothin [12], carbapenem [13], etc., has also been noted. More than half the reported strains demonstrate resistance to more than just one antibiotic [10]. Some strains have been reported to carry the mcr-1 gene responsible for resistance to the last-resort antibiotic colistin [10,14].

The availability of bacterial genomes in bulk has paved the way for bacterial pan-genomics [15,16], and coupling this approach with other features, we can study genome fraction-based traits [15,16,17,18], e.g., if a certain fraction of the genome (core, pan or accessory genome) has more or less resistance/virulence characteristics. In this study, we identify resistance genes in genome fractions and then utilize the conserved region of the genome for drug target mining against this pathogen. The conserved core region of the pathogenic genome has previously been validated using a transposon insertion sequencing approach and utilized for drug target mining in *Pseudomonas aeruginosa* [19]. The in silico approach has been utilized for resistome analysis in genome fractions and target mining from the core region in *Campylobacter* spp. [20,21]. Conventional drug discovery systems have various drawbacks, such as being costly and time-consuming. Currently, computational approaches are among the most successful choices for the development of novel vaccines and drug targets against harmful bacterial pathogens [22,23]. Therefore, we utilized this approach for inferring therapeutic targets in *E. albertii*. Additionally, virtual screening was employed to filter the best compounds from several plant compound libraries against the selected drug target, i.e., cell division protein ZipA, in order to identify natural product-based treatment for diarrhea caused by *E. albertii*. Virtual screening is a promising technique, for dealing with large libraries of molecules [24] and can be utilized for browsing databases for molecules fitting either an established pharmacophore model or a three-dimensional (3D) structure of a macromolecular target [25,26,27]. Naturally derived compounds are among the most favorable source of drug candidates to overcome antimicrobial resistance [28]. Importantly, the search for new lead structures from nature must be a priority to overcome the drying up of the antibiotic treatments pipeline against bacteria as well as the menace of multidrug resistance. Previously, such discoveries have been made against *Vibrio cholera* [29], *Shigella sonnei* [30], *Yersinia pseudotuberculosis* [31], etc. Computational assessments of Absorption, Distribution, Metabolism, Elimination and Toxicity (ADMET) work on the principle of ‘fail fast, fail early’ and save time and money for drugs that would later show adverse effects after hitting clinical trials. This is why ADMET and physiologically based pharmacokinetic (PBPK) modeling was also attempted to study time-dependent accumulation of the selected compounds in the plasma of healthy, cirrhotic and renally impaired individuals. This approach of body accumulation and retention of PBPK-informed drug development helps address knowledge gaps, boost benefit/risk assessment and deduce a dose in a healthy and diseased population. It is anticipated that the study will add to the knowledgebase of phytochemical inhibitors against the emerging pathogenesis of *E*. *albertii*.

## 2. Material and Methods

In the present study, a pan-genomic analysis-based subtractive genomic approach was applied to evaluate the novel potential drug targets and screen drug-like compounds against *E. albertii*. The detailed steps are discussed below.

### 2.1. Pan-Genomics and Core Genome Analysis

Annotated data of 95 assembled genomes of *E. albertii* from the NCBI database were downloaded and subjected to pan-genome analysis using BPGA software [32]. Pan-genome statistics were calculated according to the previously mentioned methodology [33]. Orthologs were identified with a similarity of 70% or more. Upon the addition of each new genome in the analysis pipeline, 95 random permutations of genomes were carried out to circumvent any bias. This tool fits the power-law regression model on the pan-genome data and an exponential curve fit model on core genome data. Hence, various genome fractions were identified. Clusters of orthologous groups (COG) distribution was performed to link coding DNA sequences (CDSs) to a particular class of biological function. Antibiotic resistance of the genome fractions was also profiled using the CARD database [34].

### 2.2. Prediction of Drug Targets

Core genes were subjected to the drug target mining through a subtractive approach. First of all, paralogous sequences were removed with the CD-HIT program (exclusion criteria: 60% cutoff identity). Essential genes convergent to both CEG [35] and DEG [36] databases with E-value ≤ 10^−10^ and a bit score of 100 were retained. DEG retains proteins from experimental datasets, while CEG derived its datasets from DEG, but additionally assigned data to clusters with reference to their KEGG or COG categories [35].

Translated gene products, i.e., non-homologous protein sequences to the human host (with a threshold of E-value > 0.005) and gut flora (E-value > 10^−4^), were sifted out using protein BLAST v 2.2.31. Drug targets were prioritized with an E-value < 10^−3^. A gap extension penalty of 1 and a gap penalty of 11 were considered as standard. Differential analyses were performed for 83 different species of human microbial flora in order to evaluate the novelty of our targets in the sense that they were not depicting any similarity to normal gut flora [31]. Based on extensive research, an E-value cut-off of 10^−2^ was adopted to differentiate non-homologous proteins [37]. The main purpose of this was to avoid the cross-reactivity of lead drug molecules against the proteins of normal gut flora as well as the human proteome. Only proteins non-homologous to the human host and normal gut flora proteins were selected for further investigation. Druggability screening was then carried out against latest version of the DrugBank (https://go.drugbank.com/ (accessed on 1 September 2022)), released on 3 January 2021.

### 2.3. Structural Modeling and Virtual Screening Studies

Modeling was carried out for shortlisted drug target ZipA, using a fold recognition or threading-based approach implemented on the I-TASSER server [38,39]. Generated files were fixed using PDB standards at the wwPDB validation server (https://validate-rcsb-3.wwpdb.org/ (accessed on 8 September 2022)). PDBSum tool (https://gitlab.ebi.ac.uk/roman/pdbsum1 (accessed on 8 September 2022)) was used for topology inference and ZLab for Ramachandran plot analysis (https://zlab.umass med.edu/bu/rama; accessed on 8 September 2022).

Pre-docking protonation was conducted using Molecular Operating Environment (MOE v2019 by Chemical Computing Group ULC) software with the following parameters: Atoms: all atoms; Titrate: all atoms; Flip: all atoms; Temperature = 300 K; pH = 7; Salt: 0.1; Disconnected metal treatment: enabled; Electrostatics: GB/VI with Cutoff (A): 15, Solvent: 80, Dielectric: 1; van der Waals: 800R3 with cutoff (A): 10; Protect = none. Energy minimization parameters were as follows: Forcefield: Amber99; Gradient: 0.05; Fix hydrogen and partial charges = yes. Traditional Chinese medicine (n = 36,043 compounds), and Ayurvedic compound (n = 2103 compounds) databases were taken and prepared using protonation and energy minimization of ligands. The screening was conducted using the triangle matching method, while rescoring of best pose was evaluated via London dG, which is an estimate of the free binding energy of the ligand from a given pose. Forcefield-based refining was carried out with parameters pocket cut-off of 6; final gradient: 0.01; GB/VI scoring: enabled; maximum iterations: 500; force constant: 10; and radius offset: 0.4 in pharmacophore restraint. Second rescoring was conducted using Affinity dG with the following parameters: hydrogen bond = −0.66; hydrophobic contact = −0.012; Ionic contact = 1; Hydrophobic-Polar = 0.02; Metal ligation = −1; and atom–atom value: −0.008. Duplicates were removed and only supreme complexes were retained. Results were then visualized using MOE and PyMOL (DeLano, CA, USA, 2002).

### 2.4. Dynamic Simulation Studies

MM/PBSA values were calculated for proteins, ligands, and combined complexes using the previously described methodology [31]. To deeply understand the complex interaction and stability, the high-scoring compound selected from each dataset of ligands was subjected to dynamic simulation. For this purpose, Desmond, from Schrodinger LLC software, was employed. For the verification of geometries and subsequent energy minimization, the OPLS3e forcefield was utilized. The following parameters were used to build the Desmond system: Box size assessment using a buffer (a, b, c distance = 10 Å each); a fully developed water solvation model: TIP3P; and Boundary parameters: orthorhombic box shape. Na^+^ ions were introduced for the neutralization of complexes. In the presence of Na^+^ and Cl^−^ ions, 0.15 M was the salt concentration. The simulation was run for 20 ns consisting of a recording interval of 50 ps and an energy of 5. NPT was considered as an ensemble class, with a pressure of 1.01325 bar and a temperature of 300 K (Muhammad et al., 2021). Post-simulation analysis involved the evaluation of the interactions.

### 2.5. Pharmacokinetics of Shortlisted Drug Candidates

Shortlisted compounds were checked for their computational pharmacology and pharmacokinetic properties such as ADME via SwissADME (Daina et al., 2017) and pkCSM tool (http://biosig.unimelb.edu.au/pkcsm/ (accessed on 18 September 2022)) to find out the possible best drug candidates possessing higher penetration and resulting in minimum side effects to humans. Skin permeation values were obtained from SwissADME while drug tolerance values of various organisms, including humans, were obtained from the pkCSM server. Drug safety assessment is a central issue in the new drug discovery pipeline. Ascertainment of potent drug toxicity and side effect in the early stages of drug discovery is paramount in reducing the cost and time [40,41,42]. Therefore, the parameters maximum tolerated dosage, impact on various organisms and excretion of the drug were also assessed. Simulation of the pharmcokinetic parameters in the body was conducted via compartmental model in the Gastro Plus (version 9.8.2, Simulation Plus, Inc., Lancaster, PA, USA). An oral intake of 100 mg tablet of the compound was simulated for 10 h in human body in a physiological state of fasting (population size: 250 individuals of healthy state, 250 renally impaired and 250 hepatically impaired/cirrhotic; age: 20–80 years; pH: 7.2) using a central compartment model. Particle radius was kept 25 µ and density at 1.2 g/mL, while Pka values of the compounds were determined using inbuilt ADMET profiler version 6.2. Bile salt effect was taken into account using the dissolution model of Johnson based on the Nernst–Bruner dissolution equation modified by Lu, Frisela and Johnson [43] to account for the dissolution and diffusion of spherical particles of the compound. The first pass for liver was kept fixed. Jejunal as well as a separate paracellular permeability model with the Zhimin diffusion co-efficient was included (taking into consideration the ellipsoidal shape of the molecule, represented by two molecular radii: *r_s_* and *r_he_*). A persistent electrical potential gradient was assumed for the intestinal tract length. Variables were for scaling intestinal compartments, jejunal and transcellular permeabilities. Only one source species was used, and then the bioavailablity, absorption, plasma concentration, etc., of the drug, was calculated to aid dosing.

## 3. Results

### 3.1. Pan-Genomics and Resistome Evaluation

**Figure 1:**
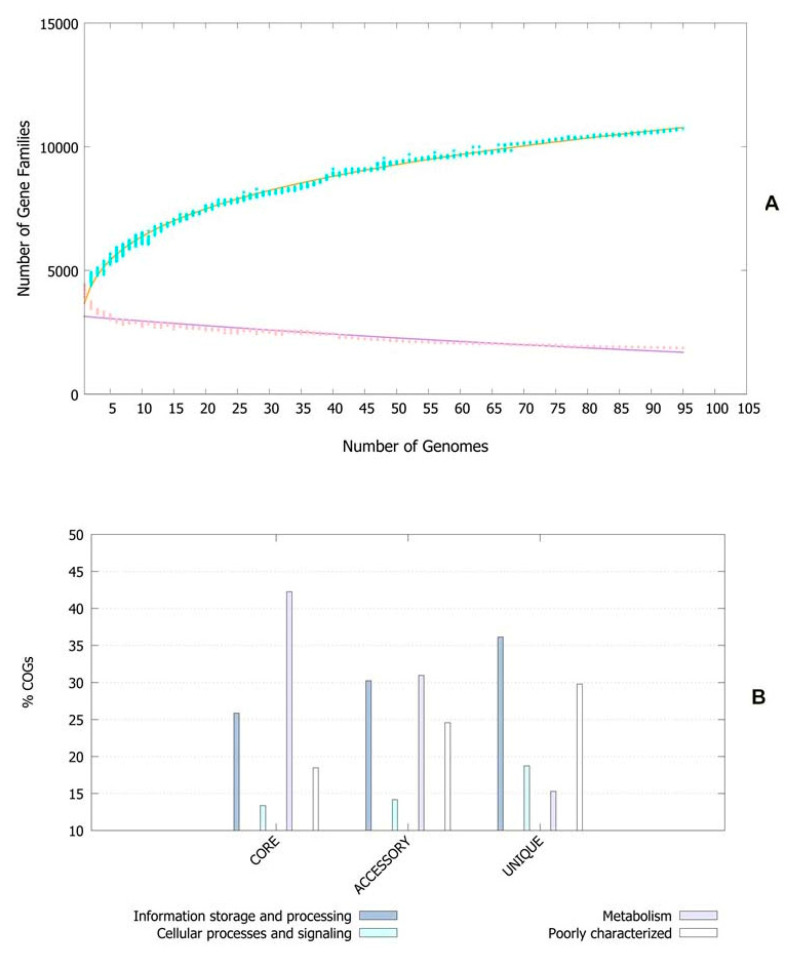
(**A**) Core–pan plot depicting the number of gene families with reference to genome fraction. (**B**) COG distribution of genes related to various processes in genome fractions of *E. albertii*.

**Figure 2:**
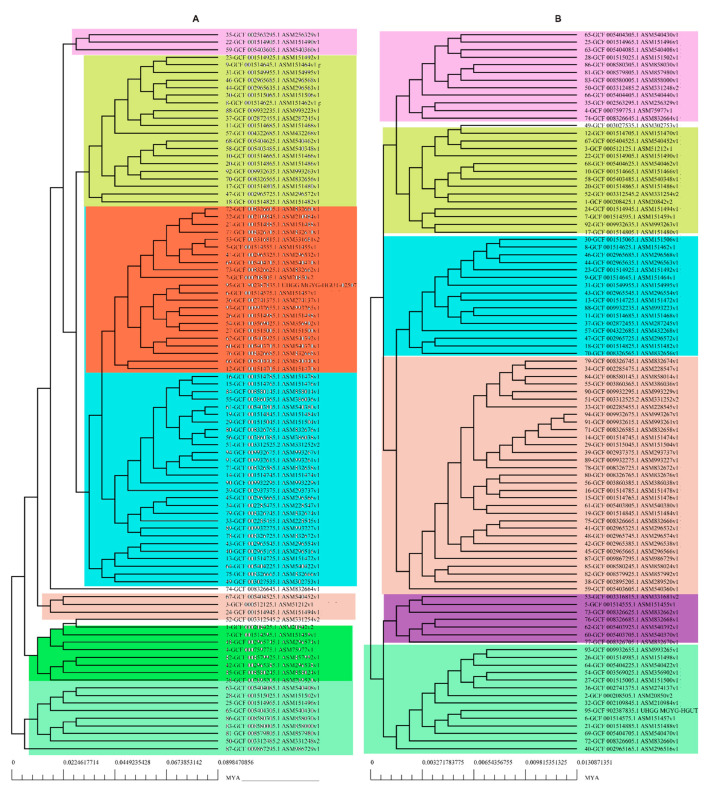
Phylogenetic profiling of (**A**) pan-genome and (**B**) core Genome of *E. albertii*.

The pan-genomic analysis resulted in the identification of a core genome having less than 2000 genes (n = 1863). The core genome accounted ofr 0.86% (1863/2,16,586 CDS) of the accessory genome fraction. The greatest number of accessory genes were found in the strain NIAH_Bird-16 (n = 2592), while the least amount was in the strain MOD1-EC1698 (n = 1924). Mkr3964 carried the greatest number of unique genes (n = 247) while the strain YS-F14-1c was found to have no such gene. Additionally, 13 strains did not have any exclusively absent gene (strain NIAH_Bird_16, EC03-195, K7756, NIAH_Bird_3, CB9786, 2014C-4356, MOD1-EC6145, 06-3542, ZAH-1-3, ZAI-5-1, G-3-3al, U-30-1, FCI-EC447). MOD1-EC5952 depicted the maximum number of absent genes, i.e., 68. Implementation of the power fit curve equation revealed that the pan-genome is plateauing and may close in the future (Figure 1A). The core genome had the highest number of metabolism-related proteins, followed by the number of information storage/processing; the number of cellular processing/signaling genes was the lowest. The unique genome had the highest amount of uncharacterized or poorly characterized content. The ratio of translational, ribosomal structure and biogenesis-related genes was highest in the core genome. The unique genome had a high range of cell/membrane or envelope biogenesis genes, which means that the outer surfaces of the bacterial strains were subjected to horizontal gene transfer. Cell division, nucleotide transport, ribosomal machinery and transport-related genes were the lowest in the accessory genome (Figure 1B). The phylogenetic analysis performed for the pan and core genomes by the BPGA tool indicated different clustering patterns of strains based on these fractions of the genome. The evolutionary distance between the strains also varied in both fractions (Figure 2).

Moreover, in the core genome, 18 genes were associated with antimicrobial resistance (AMR). Antibiotics included fluoroquinolone, nitroimidazole, peptide, macrolide, cephalosporin, triclosan, aminoglycoside, rifamycin, tetracycline, diaminopyrimidine, cephamycin and fosfomycin. Genes in the core region conferred resistance using two mechanisms, i.e., antibiotic efflux and antibiotic target alteration. A single non-synonymous mutation E350Q in the hexose phosphate transporter (UhpT) and E448K in the Glycerol-3-phosphate transporter (GlpT) was responsible for resistance to fosfomycin. Two SNPs in the gene coding for penicillin-binding protein 3 (PBP3) were found to confer resistance to cephalosporin, cephamycin and the penam class (Supplementary Table S1). Thirty-two genes were involved in AMR in the accessory genome. Nine genes (ANT(3′), CMY, TEM, APH(3″), APH(3′), APH(6), ampC-type beta-lactamase, kdpDE, resistance-nodulation-cell division (RND) protein, antibiotic efflux pump) caused resistance through the antibiotic inactivation mechanism. The class of affected antibiotics included aminoglycoside, carbapenem, cephalosporin, cephamycin, penam and monobactam. Similarly, 4 genes were associated with the replacement of antibiotics’ targets, 14 genes were involved in the antibiotic efflux mechanism and 6 genes were associated with antibiotic target alteration (Supplementary Table S2).

The unique genome comprised 13 AMR genes, i.e., APH(4), CTX-M, AAC(3), (LNU), APH(3′), AAC(3), quinolone resistance protein (qnr), ANT(2″), dfrA12, adeF, tetM, oqxA and AAC(3). These genes exhibited resistance to aminoglycoside, cephalosporin, lincosamide, fluoroquinolone, diaminopyrimidine, tetracycline and nitrofuran via antibiotic inactivation, target protection, efflux and the target replacement mechanism (Supplementary Table S3).

### 3.2. Essential Gene Prediction

In order to find the essential protein-coding genes crucial for the survival of the pathogen, we applied a hierarchical in silico approach. Essential genes are thought to be more highly evolutionary conserved than other non-essential genes [44], making them a potent drug target for therapy. This is the reason that such genes have always drawn considerable attention from researchers. The advancement in molecular techniques, especially the transposon-mediated mutagenesis approach, was a breakthrough in the discovery of essential genes [45]. Initially, the essentiality of genes was endorsed by RNA transcript inhibition and gene knock-out methods, which related to mutation insertion for loss of function in the gene. Later, databases were compiled based on such information. We used two online databases: (1) the Database of Essential Genes (DEG) and (2) the Cluster of Essential Genes (CEG). The DEG comprises ~25,000 genes from 66 different species and the CEG utilizes a prognosticating procedure with pre-determined homology-dependent clusters that demonstrate the specificity of gene activity as well as visualize conservation for the prediction of an essential gene. Essential genes for our dataset were obtained by comparing homology to sequences in both these databases. The CEG listed 1058 genes as essential to survival while the DEG came up with 1135 genes. An intersection of the genes by both tools resulted in 1041 genes as necessary for living. These essential proteins were then selected for further downstream analysis.

### 3.3. Drug Target Prediction

In order to be fully effective and cause less harm to the host, a drug is needed to selectively target the bacteria [46]. Therefore, we performed BLAST of shortlisted proteins from the CEG and DEG databases against the whole proteome of humans. We identified 532 proteins that were non-homologous to the human proteome while present in the pathogen. These shortlisted proteins for selective targeting in the pathogen. Out of these 280 proteins, 64 were found to be non-homologous to gut bacteria and 59 of these were found to be associated with virulence. Non-homologous gut sequences were further used to predict drug targets using BLAST against DrugBank sequences. Only 56 proteins matched sequences in the DrugBank, i.e., were predicted as suitable drug targets. Finally, we chose one target, i.e., ZipA, for further analysis. ZipA was considered a promising target for virtual drug screening as it is a protagonist in the cell division protein that stabilizes the FtsZ protofilaments by cross-linking them and that serves as a cytoplasmic membrane anchor for the Z ring [47].

### 3.4. Structure Modeling and Virtual Screening

**Figure 3:**
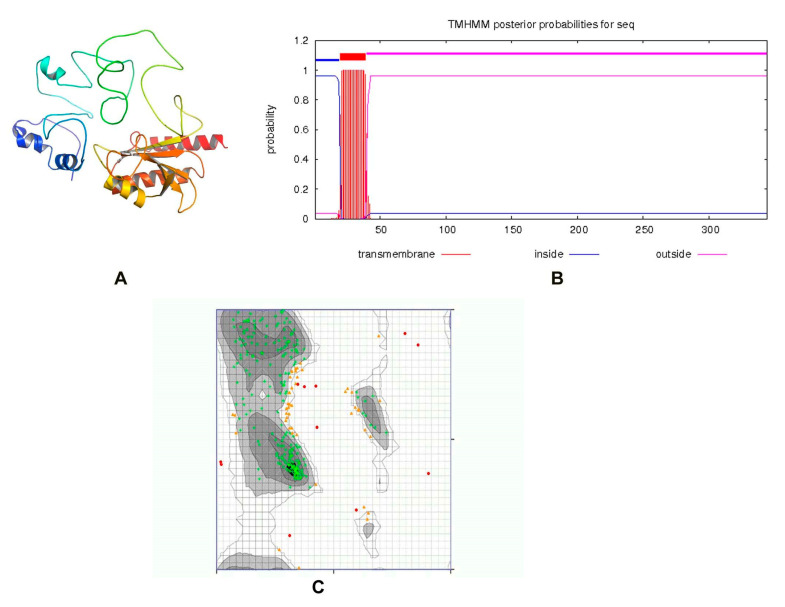
(**A**) Three-dimensional structure of ZipA. (**B**) A single transmembrane helix of 20 residues was observed (red block). The transmembrane posterior probability of ZipA is shown in red, inside-to-outside regions in blue and outside-to-inside regions in pink. (**C**) Ramachandran plot of ZipA modeled protein.

ZipA is a bitopic cytoplasmic membrane protein, having a short periplasmic N-terminal domain, a single transmembrane segment, and a large cytosolic C-terminal part [48,49]. The protein structure was modeled by the I-TASSER server, utilizing the threading approach. The top model having a TM-value 0.36 ± 0.12, C-score = −3.15, and estimated RMSD = 14.2 ± 3.8 Å was passed by ERRAT with a quality score of 75.2239, while the 3D/1D profile was ≥0.2%. The previously reported structure of ZipA in *Escherichia coli* consists of three α-helices and a β-sheet consisting of six antiparallel β-strands [50]. Visual observation showed that our predicted structure consisted of six α-helices and three β-sheets (Figure 3A). Only one transmembrane helix was present (Figure 3B). When tested with the PDBSum generate topology tool, it revealed nine α-helices, six β-strands, three beta hairpins, one beta bulge, four helix–helix interactions and 43 beta and 41 gamma turns. The protein had 79.859% amino acids in the favored region of the Ramachandran plot analysis, which indicated a fine quality, as shown in Figure 3C. This structure was used for virtual screening after energy minimization.

**Figure 4:**
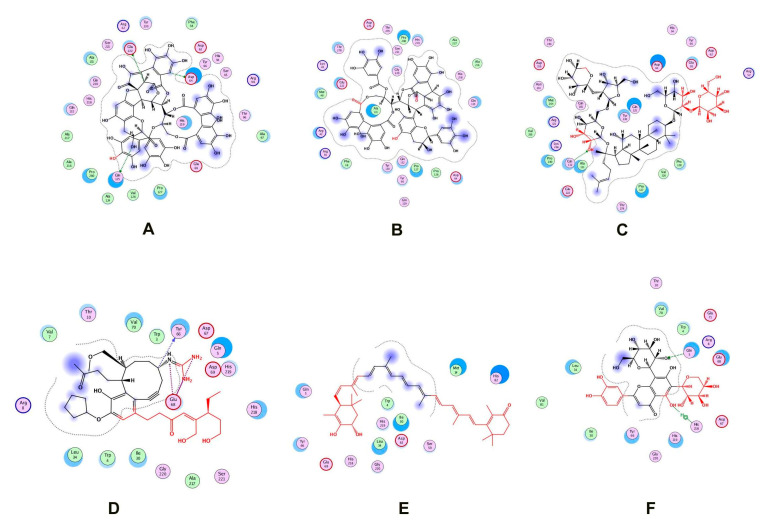
Two-dimensional interactions of (**A**) Psidinin C-Zip A complex, (**B**) ZipA-Guajavin A complex, (**C**) ZipA-Ginsenoside Ra2 complex, (**D**) ZipA-ZINC85624912 complex, (**E**) ZipA-ZINC95910716 complex and (**F**) ZipA- ZINC70450950 complex.

Two natural product libraries, the TCM and Ayurvedic compound library, were screened using the receptor-centric approach. The entire surface of the protein was screened for the best docking interactions. Docked compounds were then placed in ascending order on the basis of scoring energy values (S-score). We shortlisted only six compounds for validation: three from TCM and three from the Ayurvedic library. Among these six compounds, Psidinin C, Guajavin A and Ginsenoside Ra2 from the Ayurvedic library were chosen based on their high S-score values of −16.15, −15.63 and −15.59, respectively. Three compounds, ZINC85624912, ZINC95910716 and ZINC70450950, were selected from the TCM library, with high docking scores of −11.11, −10.49 and −10.31, respectively (Figure 4). Psidinin C and Guajavin A formed 25 interactions each. Ginsenoside Ra2 made 21 interactions, ZINC85624912 made 18 and ZINC95910716 and ZINC70450950 made 13 interactions each. Some binding site residues were shared between libraries, e.g., in Ayurvedic compound binding, Glu222, Pro280, Asp64 and Pro127 appeared in all interactions. In TCM binding residues, Leu34, Ile30, Glu68, Gly220 and His218 made an appearance in each interaction. Leu34 and Asp64 have been reported as ligand binders in *E. coli* ZipA (PBD ID: 1F46). Tyr66 was present in all the interactions, be it with TCM compounds or Ayurvedic compounds. This shows the binding tendency of certain residues towards a certain class of compounds. Tyr66 appears to be conserved, even though it was not predicted as an active site residue by I-TASSER. The detail of compounds showing hydrogen and ionic interactions are shown in Table 1. Classic simulation for obtaining MM/PBSA values showed that TCM compounds made more stable complexes compared to Ayurvedic compounds (even though MM/PBSA values of Ayurvedic compounds were lower than those of TCM compounds).

### 3.5. Molecular Dynamic Simulation Studies

In the ZipA-Psidinin C complex, the RMSD of ZipA was mostly above 3 Å, which shows that protein was undergoing large structural conformation, but it converged around 7 Å, which shows that it stabilized around a fixed value and the system was equilibrated. Ligand’s RMSD did not exceed that of protein, showing it was binding well and did not diffuse away. Psidinin C showed an RMSD of around 2 Å. Around 20 interactions were retained for more than 30% of simulation time, while 11 residues showed interactions beyond 70% of the contact time. Six residues (Arg50, Ala124, Val126, Ala131, Glu222, Met264) showed interaction beyond 90% of the simulation time. Most interactions were retained for the highest time period compared to all other ligands.

Guajavin A showed an RMSD around 2.4 Å, while ZipA showed an RMSD less than 7 Å. The complex was stable and the ligand showed binding until the end of the simulation. Complex retained seven interactions with the ligand (with Arg42, Arg50, Asp64, Tyr66, Ser221, Glu222, Thr278) for 70%, three interactions (with Arg50, Asp64, Ser221) for 90% and one interaction (with Arg50) for 100% of the simulation time. All these interactions were predicted by MOE as well. The ZipA and Ginsenoside Ra2 complex showed a stabilized RMSD between 5 and 6 Å and made five hydrogen bond/water bridge interactions at residues Glu68, Glu95, Ala97, Gln125 and Met264 for 30% of the time. One residue (Met264) showed interaction beyond 70% of the time and was present in the docked complex as well.

In the ZipA-ZINC85624912 complex, ZINC85624912 showed stabilization around 4 Å and ZipA around 5.6 Å. Ligand slightly drifted away at 17 ns but bonded again at 18 ns and retained this bonding until the end. Complex showed hydrogen bond formation with Trp3, Tyr66, Thr9, Glu68, Glu71 and His219 for more than 30% of the simulation time. The hydrogen bond with Tyr66 was also predicted by MOE. Only one hydrogen bond with Glu71 was retained for more than 70% of the simulation time with ZipA. Ile31 and Leu34 made a water bridge and Val70 showed a hydrophobic interaction for 30% of the simulation time. The Leu34 and Val70 interactions were also present during the docked stage. Pro145, Glu146, Pro147 and Pro171 of ZipA showed the highest RMSFs (>3 A).

**Figure 5:**
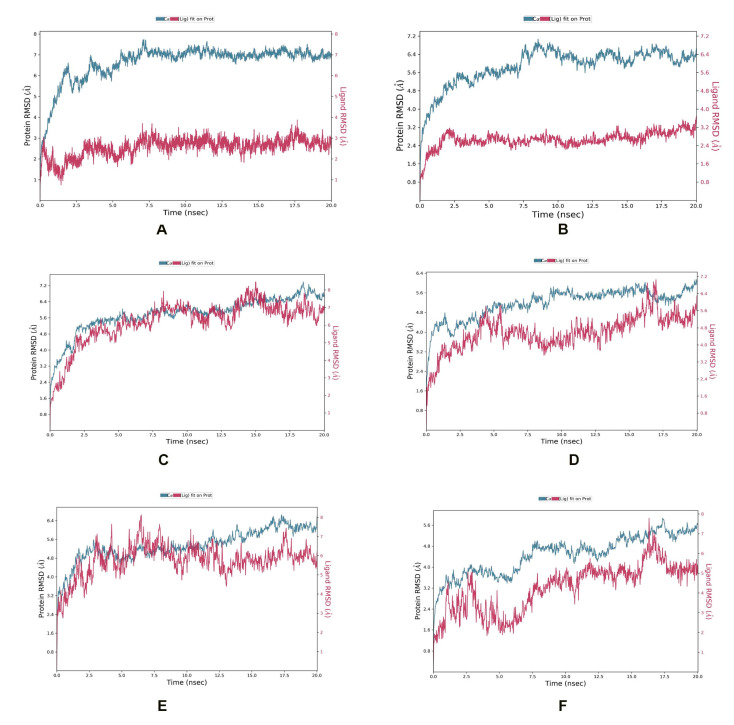
MD simulation showing RMSD for (**A**) Psidinin C-Zip A complex, (**B**) ZipA-Guajavin A complex, (**C**) ZipA-Ginsenoside Ra2 complex, (**D**) ZipA-ZINC85624912 complex, (**E**) ZipA-ZINC95910716 complex and (**F**) ZipA- ZINC70450950 complex.

ZINC70450950 showed slight drift away from protein at 4, 6 and 10 ns but later bonded and stayed attached until the end of the simulation. Protein did not show convergence at end of the simulation, and perhaps more time is required for the equilibration of this complex. Hydrogen bond and water bridge interactions were formed with Trp3, Arg8, Asp69, Glu71, and His218. The last two interactions were also seen in the docked complex. Glu71’s contact with ligand was retained for more than 70% of the simulation time. The RMSD of Zip was around 5.6 Å and around 4 Å for ZINC95910716 in the ZipA–ZINC959110716 complex. The complex also did not show convergence until the end of the simulation. It showed one hydrogen bond with Leu34, but it was not retained for longer than 30% of the simulation time. The same happened for several interactions such as Met14, Leu34, Tyr66 and Val81, which were also present in the docking results, but simulation showed non-retention for a longer time, rendering them not of interest. This shows a weaker interaction compared to ZINC85624912 and ZINC70450950, as shown in Figure 5.

Overall, Psidinin C showed the best binding interaction, and this aligns with the docking results. This compound should be further tested in vivo and in vitro for induction in the antimicrobial pipeline against *E. albertii*.

### 3.6. Pharmacokinetics of Lead Compounds

All of these compounds were substrates for p-glycoprotein, whereas none of them showed blood–brain barrier permeability (BBB) or mutagenicity. Gastrointestinal permeation was low, and these compounds did not show any inhibition against the cytochromes CYP2C9, CYP2D6, CYP2C19, CYP3A4 or CYP1A2, except for ZINC85624912 and ZINC95910716. These five compounds, except ZINC70450950, were substrates for P-glycoprotein, which means they could be disposed to efflux. Not bonding with cytochromes suggests that cytochrome is not the site of metabolism and that these compounds may be metabolized by some other proteins. The molecular polar surface area (PSA) is a very useful parameter for the prediction of drug transport properties. Polar surface area is defined as a sum of the surfaces of the polar atoms (usually oxygen, nitrogen and attached hydrogens) in a molecule. Values for this parameter, as well as other variables of ADMET of selected molecules, are shown in Table 2. ZINC85624912 showed the highest bioavailability and hence the highest absorbed fraction of the drug (Table 3). Except for ZINC95910716, all compounds showed an increased area under the curve for plasma concentration in cirrhotic and renally impaired patients compared to people not suffering from these maladies. Plasma concentration also seemed to be linked with an absorbed fraction of the drug. The minimum time to reach Cmax was observed for Ginsenoside Ra2 in the healthy patients and ZINC95910716 for the cirrhotic and renally impaired patients. Fa values depicting absorbed drug and dose movement from the lumen into the enterocytes were highest for ZINC85624912, followed by ZINC70450950. For compounds experiencing exsorption, this number may go up to a maximum and then back to lower values as the simulation is running. However, we focused on the net absorption, obtained as mean values at the end of simulation. The dose reaching the portal vein was similar to Fa values for all compounds, except for ZINC95910716. It had same Fa and FDp values, which means the absence of gut metabolism and accumulation in enterocytes. Bioavailability ‘F’ values were similar to FDp values for all compounds in diseased people, depicting a complete lack of liver metabolism in diseased conditions where renal and hepatic clearance parameters were altered.

Toxic drug effects are mostly defined as including toxicity, teratogenicity, neurotoxicity and immunotoxicity, mutagenicity and carcinogenicity. The shortlisted molecules were checked for their toxicity, and the results showed that the shortlisted molecules are not mutagens, as negative values for the AMES test were obtained. ZINC70450950 showed maximum dose tolerance in humans (Table 4), while other TCM compounds showed the least tolerance. *T. pyriformis* toxicity was highest for ZINC95910716 and similar for the rest of the compounds. All the compounds showed non-hepatotoxicity in humans, except ZINC85624912, while skin sensitization was negative for all the compounds, using neural network-based prediction. Skin sensitization and permeability values are not in compliance with the results of SwissADME, which shows different approaches may have different outcomes and need to be interpreted with caution.

## 4. Discussion

*E. albertii* is responsible for diarrhea and foodborne infections from an etiological standpoint [2]. This species diverged from *Escherichia* and some Shiga toxin-producing strains. A comparison of 2484 codon positions in 14 genes by [51] revealed that *E. albertii* strains differ, on average, at ~7.4% of the nucleotide sites from pathogenic *E. coli* strains. Ooka et al. [3] reported that the sizes of the *E. albertii* genomes range from 4.5 to 5.1 Mb, smaller than those of *E. coli* strains. Intragenus genomic comparison of 34 isolates by the group revealed that the core genome of *E. albertii* comprised 3250 genes. With an increase in the number of isolates, i.e., 95 strains, the core genome’s size decreased to less than 2000. This shows that the core genome is plateauing and the pan-genome might also be closed soon. The phylogenetic tree in our analysis did not show a specific pattern of clustering of the genomes. Intra-genome comparison of the antibiotic-resistant genes showed few genes common to two fractions of the genome. An important AMR gene, mcr-1, was detected in the accessory and unique fractions while the majority of genes were just present in one fraction and absent from the others. Li et al. [10] have previously reported the occurrence of this gene in *E. albertii* genomes, showing that it is resistant to last-resort antibiotics for multi-drug-resistant pathogens. This highlights the importance of finding new drugs against this pathogen.

Analysis of the core genome is advantageous in determining conservation status and is useful for the study of preserved essential genes in a species. These genes, if absent in humans and gut microbes, are useful as drug targets. Out of 56 drug targets from this fraction, the ZipA protein was chosen for further assessment because it is predominately involved in cell division and is essential for pathogenic proliferation. Svanberg Frisinger et al. [52] recently reported it as an essential drug target in the hyper-virulent *E. coli* strain ST131. We modeled its structure, which depicted variation from *E. coli*’s structure. This was used for screening phytochemicals. Six compounds were then shortlisted from the screened TCM and Ayurvedic databases, based on good docking scores. The results of ADMET analysis show that all six compounds could be used as lead molecules. Some residues are bound with ligands of just one library. Molecules violating Lipinski’s rule or showing toxicity could be optimized via a change in chemistry. PBPK modeling is invaluable for drug discovery and development. At the discovery stage, it is probed for initial ‘in human’ pharmacokinetics extrapolation, effect valuation, and preclinical modeling. Previously, Pepin et al. [53] compared in silico and in vitro dissolved ZURAMPIC (lesinurad) tablets on their in vivo performance, using GastroPlus. The results of Cmax values (plasma retention) were comparable to the clinical trial. Gao et al. reported experimental Cmax, Tmax and AUC values in the model organisms within twofold range of GastroPlus predicted values in humans for an inhibitor of erectile dysfunction [54]. In yet another study using GastroPlus, researchers inferred optimal dose and bioavailability for an antiviral drug Andrographolid [55] and Ticagrelor in acute coronary syndrome affected individuals (depicting liver cirrhosis), with less than a two-fold error [56]. Researchers also predicted feasible results for an antibiotic Ertapenem [57] and an adjunctive seizure treatment drug Pregabalin [58] with less than a two-fold error for renally impaired people. Therefore, we combined the healthy and diseased (renal impairment, cirrhosis/liver impairment) conditions in a population set of 250 people for each condition and used this software for the PBPK modeling of a 100 mg dose of our prioritized compounds in fasting state. A maximum plasma concentration was noted for the ZINC70450950 compound, followed by that of Guajavin A, in diseased people. Our predicted values could be taken as a guide to scale up the simulation and hence finalize dosage before laboratory experimentation. Bioavailability, plasma concentration, time to reach maximum plasma concentration and AUC were all that were altered in both healthy and diseased states. Information obtained for the prioritized compounds in this study and relevant similar studies could help in scaling dose, adjusting pH, particle size, etc., to achieve better results. PBPK modeling is prone to proliferation in the future, especially with better upcoming software. We propose that it should be made an integral part of drug design studies.

MD simulations of these compounds were also performed in complex with ZipA in order to better understand the stability and complicated interaction of selected compounds. The results highlighted the stability of protein and all shortlisted compounds after just 10 ns. Although docking showed contact of Tyr66 with all ligands, simulation showed that it was transitory in most complex interactions because it was retained for >30% of simulation time for just Guajavin A and ZINC85624912, not for the other four ligands. On the whole, our analysis showed that complexes were stable and, along with their useful properties, have the potential to be introduced inin the drug pipeline against *E. albertii*.

## 5. Conclusions

Pathogens are particularly efficient at generating antibiotic resistance because they acquire mutations very rapidly, making it more challenging for traditional drug development strategies to cope with the rate of resistance evolution. The main advantage of the subtractive genomic technique used in this study is the ability to find selective targets that impact the pathogen while remaining safe for the host and gut bacteria. The virtual screening method is a quick way to filter out therapeutic compounds from huge libraries that could effectively work against these pathogens. We were able to find compounds that could target pathogenic ZipA protein and, hopefully, avoid cross-reactivity with host proteins, reducing the risk of problems following drug administration. MD simulations were also performed, and the findings revealed that Psidinin C had the best binding interaction, which corresponded to the docking data. The ADMET profiling of the best-docked compounds helped find properties that could further rank drug usefulness and toxicity. These showed that ZINC70450950 was tolerated by humans the best. However, healthy and organ-impaired PBPK modeling showed that ZINC85624912 had the highest bioavailability and plasma retention in healthy and hepatic and renally impaired populations. The selected compounds need to be further evaluated, modified if necessary and then tested in vitro and in vivo for inclusion in the antimicrobial pipeline against *E. albertii*.

## Figures and Tables

**Table 1:** Interaction distance and energy values for the top docked complexes. MM/PBSA value of the protein was −40.22.

Library	Name	MM/PBSA of Ligand	MM/PBSA of Protein-Ligand Complex	S-Value	Atoms	Interaction	Bond Length (Å)	Energy kcal/mol
Ayurvedic	Psidinin C	−0.79	−39.39	−16.15	O34 with Gln125 O76 with Glu222 C82 with Asp64	H-donor H-donor H-donor	2.59 3.12 2.82	−2.8 −0.8 −1.6
	Guajavin A	−0.91	−39.32	−15.63	-	-	-	-
	Ginsenoside Ra2	−1.28	−39.06	−15.59	O66 with Gln133	H-acceptor	2.86	−1.9
TCM	ZINC85624912	−0.58	−39.62	−11.11	C22 with Tyr66 N35 with Glu68 N37 with Glu68 N38 with Glu68	H-donor Ionic bond Ionic bond Ionic bond	3.14 2.77 2.57 3.34	−0.5 −6.2 −8.2 −2.6
	ZINC95910716	−0.73	−39.64	−10.49	-	-	-	-
	ZINC70450950	−0.16	−39.85	−10.31	O28 with Gln5 O39 with His218	H-acceptor H-pi	2.36 4.38	−0.9 −1.9

**Table 2:** Pharmacokinetic parameters of the six top-scoring compounds.

Library	Compound	Molar Refractivity	Total PSA (Å^2^)	Bioavailability Score	Lipinski Violations	Lead Likeness Violation	Consensus Log P	Skin Permeation (cm/s)
Ayurvedic	Psidinin C	275.45	531.17	0.17	3	1	0.01	−12.29
	Guajavin A	285.74	565.56	0.17	3	1	0.17	−11.68
	Ginsenoside Ra2	289.86	415.98	0.17	3	2	−1.44	−14.14
TCM	ZINC85624912	186.68	179.30	0.17	2	2	3.60	−8.05
	ZINC95910716	185.99	54.37	0.17	2	2	3.60	−8.05
	ZINC70450950	135.28	271.20	0.17	3	1	−2.12	−11.64

**Table 3:** Parameters of the drug absorption kinetics in human host.

Library	Absorbed Fraction of the Drug Fa [%]	Dose Reaching the Portal Vein FDp [%]	Bioavailability F [%]	Maximum Plasma Concentration Cmax [ug/mL]	Time of Cmax [h]	Absorbed Fraction of the Drug Fa [%]	AUC(0-inf) [ng-h/mL]	AUC(0-t) [ng-h/mL]
Ayurvedic	Psidinin C in healthy	0.9879	0.6885	0.6305	0.0058	9.5444	327.24	28.243
	Psidinin C in CP	0.8983	0.6273	0.6273	0.0472	10	214.82	214.82
	Psidinin C in RI patient	0.908	0.6516	0.6516	0.051	10	239.97	239.97
	Guajavin A in healthy	2.5604	1.7364	1.4894	0.0147	8.3008	571.27	86.599
	Guajavin A in CP	2.3592	1.5299	1.5299	0.1175	10	556.53	556.53
	Guajavin A in RI patient	2.4877	1.5906	1.5906	0.1227	10	558.26	558.26
	Ginsenoside Ra2 in healthy	5.0919	3.091	1.8541	0.0111	6.1048	195.96	71.161
	Ginsenoside Ra2 in CP	5.2242	3.0946	3.0946	0.0575	10	280.97	280.97
	Ginsenoside Ra2 in RI patient	5.335	3.2377	3.2377	0.0699	10	347.9	347.9
TCM	ZINC85624912 in healthy	50.29	47.188	45.071	0.1246	9.1224	43220	947.63
	ZINC85624912 in CP	47.613	44.505	44.505	0.2587	8.9028	14570	2122.5
	ZINC85624912 in RI patient	49.613	46.628	46.628	0.2701	6.3164	184500	2189.2
	ZINC95910716 in healthy	3.158	3.1571	3.1558	0.0196	10	146.32	146.32
	ZINC95910716 in CP	3.2646	3.2638	3.2638	0.0089	2.1232	90.23	48.93
	ZINC95910716 in RI patient	2.9727	2.9716	2.9716	0.01	2.5384	101.83	56.385
	ZINC70450950 in healthy	13.043	11.493	10.761	0.1502	6.9704	6414.3	1086.5
	ZINC70450950 in CP	13.471	11.975	11.975	0.5176	9.9728	45950	3424.7
	ZINC70450950 in RI patient	14.462	12.693	12.693	0.5496	9.9792	10900	3520.5

AUC(0-inf) [ng-h/mL] = Area under the plasma concentration–time curve in the central compartment extrapolated to infinity; AUC(0-t) [ng-h/mL] = Area under the central compartment for plasma concentration–time curve during time of simulation.

**Table 4:** Toxicity profile of the six top-scoring compounds.

Library	Compound	Maximum Tolerated Dose in Humans (log mg/kg/day)	Oral Rat Acute Toxicity (LD50) (mol/kg)	Oral Rat Chronic Toxicity (LOAEL) (log mg/kg_bw/day)	*T. pyriformis* Toxicity (log ug/L)	Minnow Toxicity (log mM)
Ayurvedic	Psidinin C	0.438	0.438	17.76	0.285	12.923
	Guajavin A	0.438	2.482	21.14	0.285	21.762
	Ginsenoside Ra2	0.231	2.494	6.779	0.285	13.367
TCM	ZINC85624912	−0.351	2.679	1.812	0.285	2.278
	ZINC95910716	−0.438	2.171	2.468	0.342	−3.178
	ZINC70450950	0.473	2.472	6.616	0.285	7.377

## Data Availability

No new data were generated in this work that require submission in a repository. Details of the data used in this research are included within the manuscript.

## Supplementary Materials

The following supporting information can be downloaded at: https://www.mdpi.com/article/10.3390/life13020541/s1. Table S1: Antibiotic resistance gene from the core genome fraction; Table S2: Antibiotic resistance gene from the accessory genome fraction; Table S3: Antibiotic resistance gene from the unique genome fraction.

## References

[1] Lima M.P., Yamamoto D., Santos A.C.M., Ooka T., Hernandes R.T., Vieira M.A.M., Santos F.F., Silva R.M., Hayashi T. (2019). Gomes TAT: Phenotypic characterization and virulence-related properties of Escherichia albertii strains isolated from children with diarrhea in Brazil. Pathog. Dis..

[2] Masuda K., Ooka T., Akita H., Hiratsuka T., Takao S., Fukada M., Inoue K., Honda M., Toda J. (2020). Sugitani W: Epidemiological aspects of Escherichia albertii outbreaks in Japan and genetic characteristics of the causative pathogen. Foodborne Pathog. Dis..

[3] Ooka T., Ogura Y., Katsura K., Seto K., Kobayashi H., Kawano K., Tokuoka E., Furukawa M., Harada S., Yoshino S. (2015). Defining the genome features of Escherichia albertii, an emerging enteropathogen closely related to Escherichia coli. Genome Biol. Evol..

[4] Inglis T.J., Merritt A.J., Bzdyl N., Lansley S., Urosevic M.N. (2015). First bacteraemic human infection with Escherichia albertii. New Microbes New Infect.

[5] Albert M.J., Alam K., Islam M., Montanaro J., Rahaman A., Haider K., Hossain M.A., Kibriya A., Tzipori S. (1991). Hafnia alvei, a probable cause of diarrhea in humans. Infect. Immun..

[6] Huys G., Cnockaert M., Janda J.M., Swings J. (2003). *Escherichia albertii* sp. nov., a diarrhoeagenic species isolated from stool specimens of Bangladeshi children. Int. J. Syst. Evol. Microbiol..

[7] Asoshima N., Matsuda M., Shigemura K., Honda M., Yoshida H., Hiwaki H., Ogata K., Oda T. (2014). Identification of Escherichia albertii as a causative agent of a food-borne outbreak occurred in 2003. Jpn. J. Infect Dis..

[8] Hinenoya A., Yasuda N., Mukaizawa N., Sheikh S., Niwa Y., Awasthi S.P., Asakura M., Tsukamoto T., Nagita A., Albert M.J. (2017). Association of cytolethal distending toxin-II gene-positive Escherichia coli with Escherichia albertii, an emerging enteropathogen. Int. J. Med. Microbiol..

[9] Perez K.L., Alam M.J., Castillo A., Taylor T.M. (2013). Antibiotic resistance and growth of the emergent pathogen Escherichia albertii on raw ground beef stored under refrigeration, abuse, and physiological temperature. J. Food Prot..

[10] Li Q., Wang H., Xu Y., Bai X., Wang J., Zhang Z., Liu X., Miao Y., Zhang L., Li X. (2018). Multidrug-Resistant Escherichia albertii: Co-occurrence of beta-Lactamase and MCR-1 Encoding Genes. Front Microbiol..

[11] Hinenoya A., Wang H., Patrick E.M., Zeng X., Cao L., Li X.P., Lindsey R.L., Gillespie B., He Q., Yamasaki S. (2022). Longitudinal surveillance and comparative characterization of Escherichia albertii in wild raccoons in the United States. Microbiol. Res..

[12] Hinenoya A., Li X.P., Zeng X., Sahin O., Moxley R.A., Logue C.M., Gillespie B., Yamasaki S., Lin J. (2021). Isolation and characterization of Escherichia albertii in poultry at the pre-harvest level. Zoonoses Public Health.

[13] Wang H., Zhang L., Cao L., Zeng X., Gillespie B., Lin J. (2022). Isolation and characterization of Escherichia albertii originated from the broiler farms in Mississippi and Alabama. Vet. Microbiol..

[14] Sonnevend A., Alali W.Q., Mahmoud S.A., Ghazawi A., Bharathan G., Melegh S., Rizvi T.A., Pal T. (2022). Molecular Characterization of MCR-1 Producing Enterobacterales Isolated in Poultry Farms in the United Arab Emirates. Antibiotics.

[15] Gonzales-Siles L., Karlsson R., Schmidt P., Salva-Serra F., Jaen-Luchoro D., Skovbjerg S., Moore E.R.B., Gomila M. (2020). A Pangenome Approach for Discerning Species-Unique Gene Markers for Identifications of Streptococcus pneumoniae and Streptococcus pseudopneumoniae. Front Cell Infect Microbiol..

[16] Liao J., Guo X., Weller D.L., Pollak S., Buckley D.H., Wiedmann M., Cordero O.X. (2021). Nationwide genomic atlas of soil-dwelling Listeria reveals effects of selection and population ecology on pangenome evolution. Nat. Microbiol..

[17] Golanowska M., Potrykus M., Motyka-Pomagruk A., Kabza M., Bacci G., Galardini M., Bazzicalupo M., Makalowska I., Smalla K., Mengoni A. (2018). Comparison of Highly and Weakly Virulent Dickeya solani Strains, With a View on the Pangenome and Panregulon of This Species. Front. Microbiol..

[18] Sun W., Sun X., Haggblom M.M., Kolton M., Lan L., Li B., Dong Y., Xu R., Li F. (2021). Identification of Antimonate Reducing Bacteria and Their Potential Metabolic Traits by the Combination of Stable Isotope Probing and Metagenomic-Pangenomic Analysis. Environ. Sci. Technol..

[19] Poulsen B.E., Yang R., Clatworthy A.E., White T., Osmulski S.J., Li L., Penaranda C., Lander E.S., Shoresh N., Hung D.T. (2019). Defining the core essential genome of Pseudomonas aeruginosa. Proc. Natl. Acad. Sci. USA.

[20] Jalal K., Khan K., Hayat A., Ahmad D., Alotaibi G., Uddin R., Mashraqi M.M., Alzamami A., Aurongzeb M., Basharat Z. (2022). Mining therapeutic targets from the antibiotic-resistant Campylobacter coli and virtual screening of natural product inhibitors against its riboflavin synthase. Mol. Divers..

[21] Khan K., Basharat Z., Jalal K., Mashraqi M.M., Alzamami A., Alshamrani S., Uddin R. (2022). Identification of Therapeutic Targets in an Emerging Gastrointestinal Pathogen Campylobacter ureolyticus and Possible Intervention through Natural Products. Antibiotics.

[22] Basharat Z., Akhtar U., Khan K., Alotaibi G., Jalal K., Abbas M.N., Hayat A., Ahmad D., Hassan S.S. (2022). Differential analysis of Orientia tsutsugamushi genomes for therapeutic target identification and possible intervention through natural product inhibitor screening. Comput. Biol. Med..

[23] Aslam M., Shehroz M., Ali F., Zia A., Pervaiz S., Shah M., Hussain Z., Nishan U., Zaman A., Afridi S.G. (2021). Chlamydia trachomatis core genome data mining for promising novel drug targets and chimeric vaccine candidates identification. Comput. Biol. Med..

[24] Maia E.H.B., Assis L.C., de Oliveira T.A., da Silva A.M., Taranto A.G. (2020). Structure-Based Virtual Screening: From Classical to Artificial Intelligence. Front. Chem..

[25] Vora J., Patel S., Sinha S., Sharma S., Srivastava A., Chhabria M., Shrivastava N. (2019). Structure based virtual screening, 3D-QSAR, molecular dynamics and ADMET studies for selection of natural inhibitors against structural and non-structural targets of Chikungunya. J. Biomol. Struct. Dyn..

[26] Elseginy S.A. (2021). Virtual screening and structure-based 3D pharmacophore approach to identify small-molecule inhibitors of SARS-CoV-2 M(pro). J. Biomol. Struct. Dyn..

[27] Erlina L., Paramita R.I., Kusuma W.A., Fadilah F., Tedjo A., Pratomo I.P., Ramadhanti N.S., Nasution A.K., Surado F.K., Fitriawan A. (2022). Virtual screening of Indonesian herbal compounds as COVID-19 supportive therapy: Machine learning and pharmacophore modeling approaches. BMC Complement Med. Ther..

[28] Atanasov A.G., Zotchev S.B., Dirsch V.M. (2021). International Natural Product Sciences T, Supuran CT: Natural products in drug discovery: Advances and opportunities. Nat. Rev. Drug Discov..

[29] Barh D., Barve N., Gupta K., Chandra S., Jain N., Tiwari S., Leon-Sicairos N., Canizalez-Roman A., dos Santos A.R., Hassan S.S. (2013). Exoproteome and secretome derived broad spectrum novel drug and vaccine candidates in Vibrio cholerae targeted by Piper betel derived compounds. PLoS ONE.

[30] Basharat Z., Jahanzaib M., Rahman N. (2021). Therapeutic target identification via differential genome analysis of antibiotic resistant Shigella sonnei and inhibitor evaluation against a selected drug target. Infect. Genet. Evol..

[31] Basharat Z., Jahanzaib M., Yasmin A., Khan I.A. (2021). Pan-genomics, drug candidate mining and ADMET profiling of natural product inhibitors screened against Yersinia pseudotuberculosis. Genomics.

[32] Chaudhari N.M., Gupta V.K., Dutta C. (2016). BPGA-an ultra-fast pan-genome analysis pipeline. Sci. Rep..

[33] Basharat Z., Yasmin A., He T., Tong Y. (2018). Genome sequencing and analysis of Alcaligenes faecalis subsp. phenolicus MB207. Sci. Rep..

[34] Alcock B.P., Raphenya A.R., Lau T.T., Tsang K.K., Bouchard M., Edalatmand A., Huynh W., Nguyen A.-L.V., Cheng A.A., Liu S. (2020). CARD 2020: Antibiotic resistome surveillance with the comprehensive antibiotic resistance database. Nucleic Acids Res..

[35] Ye Y.-N., Hua Z.-G., Huang J., Rao N., Guo F.-B. (2013). CEG: A database of essential gene clusters. BMC Genom..

[36] Luo H., Lin Y., Liu T., Lai F.-L., Zhang C.-T., Gao F., Zhang R. (2021). DEG 15, an update of the Database of Essential Genes that includes built-in analysis tools. Nucleic Acids Res..

[37] Sarangi A.N., Lohani M., Aggarwal R. (2015). Proteome mining for drug target identification in Listeria monocytogenes strain EGD-e and structure-based virtual screening of a candidate drug target penicillin binding protein 4. J. Microbiol. Methods.

[38] Zheng W., Zhang C., Bell E.W., Zhang Y. (2019). I-TASSER gateway: A protein structure and function prediction server powered by XSEDE. Future Gener Comput. Syst..

[39] Zhou X., Zheng W., Li Y., Pearce R., Zhang C., Bell E.W., Zhang G., Zhang Y. (2022). I-TASSER-MTD: A deep-learning-based platform for multi-domain protein structure and function prediction. Nat. Protoc..

[40] Wei M.P., Qiu J.D., Li L., Xie Y.F., Yu H., Guo Y.H., Yao W.R. (2021). Saponin fraction from Sapindus mukorossi Gaertn as a novel cosmetic additive: Extraction, biological evaluation, analysis of anti-acne mechanism and toxicity prediction. J. Ethnopharmacol..

[41] Banerjee R., Kumar M., Gaurav I., Thakur S., Thakur A., Singh K., Karak S., Das R., Chhabra M. (2021). In-silico Prediction of the Beta-carboline Alkaloids Harmine and Harmaline as Potent Drug Candidates for the Treatment of Parkinson’s disease. Antiinflamm Antiallergy Agents Med. Chem..

[42] Rai H., Barik A., Singh Y.P., Suresh A., Singh L., Singh G., Nayak U.Y., Dubey V.K., Modi G. (2021). Molecular docking, binding mode analysis, molecular dynamics, and prediction of ADMET/toxicity properties of selective potential antiviral agents against SARS-CoV-2 main protease: An effort toward drug repurposing to combat COVID-19. Mol. Divers.

[43] Lu A.T., Frisella M.E., Johnson K.C. (1993). Dissolution modeling: Factors affecting the dissolution rates of polydisperse powders. Pharm. Res..

[44] Luo J., Wu J. (2015). A new algorithm for essential proteins identification based on the integration of protein complex co-expression information and edge clustering coefficient. Int. J. Data Min. Bioinform..

[45] Luo H., Gao F., Lin Y. (2015). Evolutionary conservation analysis between the essential and nonessential genes in bacterial genomes. Sci. Rep..

[46] Shanmugham B., Pan A. (2013). Identification and characterization of potential therapeutic candidates in emerging human pathogen Mycobacterium abscessus: A novel hierarchical in silico approach. PLoS ONE.

[47] Vega D.E., Margolin W. (2019). Direct Interaction between the Two Z Ring Membrane Anchors FtsA and ZipA. J. Bacteriol..

[48] Hale C.A., Shiomi D., Liu B., Bernhardt T.G., Margolin W., Niki H., de Boer P.A. (2011). Identification of Escherichia coli ZapC (YcbW) as a component of the division apparatus that binds and bundles FtsZ polymers. J. Bacteriol..

[49] Pazos M., Natale P., Vicente M. (2013). A specific role for the ZipA protein in cell division: Stabilization of the FtsZ protein. J. Biol. Chem..

[50] Moy F.J., Glasfeld E., Mosyak L., Powers R. (2000). Solution structure of ZipA, a crucial component of Escherichia coli cell division. Biochemistry.

[51] Hyma K.E., Lacher D.W., Nelson A.M., Bumbaugh A.C., Janda J.M., Strockbine N.A., Young V.B., Whittam T.S. (2005). Evolutionary genetics of a new pathogenic Escherichia species: Escherichia albertii and related Shigella boydii strains. J. Bacteriol..

[52] Svanberg Frisinger F., Jana B., Donadio S., Guardabassi L. (2021). In Silico Prediction and Prioritization of Novel Selective Antimicrobial Drug Targets in Escherichia coli. Antibiotics.

[53] Pepin X.J., Flanagan T.R., Holt D.J., Eidelman A., Treacy D., Rowlings C.E. (2016). Justification of Drug Product Dissolution Rate and Drug Substance Particle Size Specifications Based on Absorption PBPK Modeling for Lesinurad Immediate Release Tablets. Mol. Pharm..

[54] Gao Z.W., Zhu Y.T., Yu M.M., Zan B., Liu J., Zhang Y.F., Chen X.Y., Li X.N., Zhong D.F. (2015). Preclinical pharmacokinetics of TPN729MA, a novel PDE5 inhibitor, and prediction of its human pharmacokinetics using a PBPK model. Acta Pharmacol. Sin..

[55] Talapphetsakun T., Viyoch J., Waranuch N., Sermsappasuk P. (2022). The Development of a Physiologically Based Pharmacokinetic (PBPK) Model of Andrographolide in Mice and Scaling It up to Rats, Dogs and Humans. Curr. Drug. Metab..

[56] Zhang M., You X., Ke M., Jiao Z., Wu H., Huang P., Lin C. (2019). Prediction of Ticagrelor and its Active Metabolite in Liver Cirrhosis Populations Using a Physiologically Based Pharmacokinetic Model Involving Pharmacodynamics. J. Pharm. Sci..

[57] Ye L., Ke M., You X., Huang P., Lin C. (2020). A Physiologically Based Pharmacokinetic Model of Ertapenem in Pediatric Patients with Renal Impairment. J. Pharm. Sci..

[58] Ke C., You X., Lin C., Chen J., Guo G., Wu W., Ye L., Huang P. (2022). Development of Physiologically Based Pharmacokinetic Model for Pregabalin to Predict the Pharmacokinetics in Pediatric Patients with Renal Impairment and Adjust Dosage Regimens: PBPK Model of Pregabalin in Pediatric Patients with Renal Impairment. J. Pharm. Sci..

