# Supplementary material for: Pan-Genomics of Escherichia albertii for Antibiotic Resistance Profiling in Different Genome Fractions and Natural Product Mediated Intervention: In Silico Approach
Source: Life (Basel). 2023 Feb 15;13(2):541. doi: 10.3390/life13020541 (PMC9962377; doi:10.3390/life13020541)
Supplement: Supplementary file 1 [file life-13-00541-s001.zip › Supplementary Table S1.pdf]

Supplementary Table S1. Antibiotic resistance gene from the core genome fraction.

Core Resistome								
**RGI Criteria**	**ARO Term**	**SNP**	**Detection Criteria**	**AMR Gene Family**	**Drug Class**	**Resistance Mechanism**	**% Identity of Matching Region**	**% Length of Reference Sequence**
Strict	acrB		protein homolog model	resistance-nodulation-cell division (RND) antibiotic efflux pump	fluoroquinolone antibiotic, cephalosporin, glycylcycline, penam, tetracycline antibiotic, rifamycin antibiotic, phenicol antibiotic, triclosan	antibiotic efflux	98.47	100.00
Strict	msbA		protein homolog model	ATP-binding cassette (ABC) antibiotic efflux pump	nitroimidazole antibiotic	antibiotic efflux	98.28	100.00
Strict	eptA		protein homolog model	pmr phosphoethanolamine transferase	peptide antibiotic	antibiotic target alteration	94.3	100.00
Strict	Klebsiella pneumoniae KpnH		protein homolog model	major facilitator superfamily (MFS) antibiotic efflux pump	macrolide antibiotic, fluoroquinolone antibiotic, aminoglycoside antibiotic, carbapenem,	antibiotic efflux	99.22	100.00

					cephalosporin, penam, peptide antibiotic, penem			
Strict	Escherichia coli acrA		protein homolog model	resistance-nodulation-cell division (RND) antibiotic efflux pump	fluoroquinolone antibiotic, cephalosporin, glycylcycline, penam, tetracycline antibiotic, rifamycin antibiotic, phenicol antibiotic, triclosan	antibiotic efflux	97.48	100.00
Strict	bacA		protein homolog model	undecaprenyl pyrophosphate related proteins	peptide antibiotic	antibiotic target alteration	98.9	100.00
Strict	CRP		protein homolog model	resistance-nodulation-cell division (RND) antibiotic efflux pump	macrolide antibiotic, fluoroquinolone antibiotic, penam	antibiotic efflux	99.52	100.00
Strict	emrR		protein homolog model	major facilitator superfamily (MFS) antibiotic efflux pump	fluoroquinolone antibiotic	antibiotic efflux	97.73	100.00
Strict	H-NS		protein homolog model	major facilitator superfamily (MFS) antibiotic efflux pump, resistance-nodulation-cell division (RND)	macrolide antibiotic, fluoroquinolone antibiotic, cephalosporin, cephamycin, penam,	antibiotic efflux	98.54	100.00

				antibiotic efflux pump	tetracycline antibiotic			
Strict	Klebsiella pneumoniae KpnE		protein homolog model	major facilitator superfamily (MFS) antibiotic efflux pump	macrolide antibiotic, aminoglycoside antibiotic, cephalosporin, tetracycline antibiotic, peptide antibiotic, rifamycin antibiotic	antibiotic efflux	77.97	100.83
Strict	Escherichia coli emrE		protein homolog model	small multidrug resistance (SMR) antibiotic efflux pump	macrolide antibiotic	antibiotic efflux	96.36	100.00
Strict	Klebsiella pneumoniae KpnF		protein homolog model	major facilitator superfamily (MFS) antibiotic efflux pump	macrolide antibiotic, aminoglycoside antibiotic, cephalosporin, tetracycline antibiotic, peptide antibiotic, rifamycin antibiotic	antibiotic efflux	85.32	100.00
Strict	rsmA		protein homolog model	resistance-nodulation-cell division (RND) antibiotic efflux pump	fluoroquinolone antibiotic, diaminopyrimidine antibiotic, phenicol antibiotic	antibiotic efflux	85.25	100.00
Strict	Haemophilus influenzae PBP3	D350N, S357N	protein variant model	Penicillin-binding protein mutations	cephalosporin, cephamycin, penam	antibiotic target	53.29	96.39

	conferring resistance to beta-lactam antibiotics			conferring resistance to beta-lactam antibiotics		alteration		
Strict	Escherichia coli UhpT with mutation conferring resistance to fosfomycin	E350Q	protein variant model	antibiotic-resistant UhpT	fosfomycin	antibiotic target alteration	97.84	100.00
Strict	Escherichia coli GlpT with mutation conferring resistance to fosfomycin	E448K	protein variant model	antibiotic-resistant GlpT	fosfomycin	antibiotic target alteration	97.57	100.00
Strict	Escherichia coli acrR with mutation conferring multidrug antibiotic resistance		protein overexpression model	resistance-nodulation-cell division (RND) antibiotic efflux pump	fluoroquinolone antibiotic, cephalosporin, glycylcycline, penam, tetracycline antibiotic, rifamycin antibiotic, phenicol antibiotic, triclosan	antibiotic target alteration, antibiotic efflux	94.39	100.93

Strict	Escherichia coli soxR with mutation conferring antibiotic resistance		protein overexpression model	ATP-binding cassette (ABC) antibiotic efflux pump, major facilitator superfamily (MFS) antibiotic efflux pump, resistance-nodulation-cell division (RND) antibiotic efflux pump	fluoroquinolone antibiotic, cephalosporin, glycylcycline, penam, tetracycline antibiotic, rifamycin antibiotic, phenicol antibiotic, triclosan	antibiotic target alteration, antibiotic efflux	98.7	100.00
