# Supplementary material for: Pan-Genomics of Escherichia albertii for Antibiotic Resistance Profiling in Different Genome Fractions and Natural Product Mediated Intervention: In Silico Approach
Source: Life (Basel). 2023 Feb 15;13(2):541. doi: 10.3390/life13020541 (PMC9962377; doi:10.3390/life13020541)
Supplement: Supplementary file 1 [file life-13-00541-s001.zip › Supplementary Table S2.pdf]

Supplementary Table S2. Antibiotic resistance gene from the accessory genome fraction.

Accessory Resistome								
**RGI Criteria**	**ARO Term**	**SNP**	**Detection Criteria**	**AMR Gene Family**	**Drug Class**	**Resistance Mechanism**	**% Identity of Matching Region**	**% Length of Reference Sequence**
Perfect	aadA13		protein homolog model	ANT(3')	aminoglycoside antibiotic	antibiotic inactivation	100.0	100.00
Perfect	CMY-2		protein homolog model	CMY beta-lactamase	carbapenem, cephalosporin, cephamycin, penam	antibiotic inactivation	100.0	100.00
Perfect	dfrA5		protein homolog model	trimethoprim resistant dihydrofolate reductase dfr	diaminopyrimidine antibiotic	antibiotic target replacement	100.0	100.00
Perfect	dfrA8		protein homolog model	trimethoprim resistant dihydrofolate reductase dfr	diaminopyrimidine antibiotic	antibiotic target replacement	100.0	100.00
Perfect	MCR-1.1		protein homolog model	MCR phosphoethanolamine transferase	peptide antibiotic	antibiotic target alteration	100.0	100.00
Perfect	qacEdelta1		protein homolog model	major facilitator superfamily (MFS) antibiotic efflux pump	acridine dye, disinfecting agents and intercalating dyes	antibiotic efflux	100.0	100.00

Perfect	sul1		protein homolog model	sulfonamide resistant sul	sulfonamide antibiotic	antibiotic target replacement	100.0	100.00
Perfect	sul3		protein homolog model	sulfonamide resistant sul	sulfonamide antibiotic	antibiotic target replacement	100.0	100.00
Perfect	TEM-1		protein homolog model	TEM beta-lactamase	monobactam, cephalosporin, penam, penem	antibiotic inactivation	100.0	100.00
Strict	APH(3'')-Ib		protein homolog model	APH(3'')	aminoglycoside antibiotic	antibiotic inactivation	99.63	100.00
Strict	APH(3')-Ia		protein homolog model	APH(3')	aminoglycoside antibiotic	antibiotic inactivation	98.15	100.00
Strict	APH(6)-Id		protein homolog model	APH(6)	aminoglycoside antibiotic	antibiotic inactivation	99.64	100.00
Strict	baeR		protein homolog model	resistance-nodulation-cell division (RND) antibiotic efflux pump	aminoglycoside antibiotic, aminocoumarin antibiotic	antibiotic efflux	98.74	105.83
Strict	baeS		protein homolog model	resistance-nodulation-cell division (RND) antibiotic efflux pump	aminoglycoside antibiotic, aminocoumarin antibiotic	antibiotic efflux	96.57	100.00

Strict	cmlA1		protein homolog model	major facilitator superfamily (MFS) antibiotic efflux pump	phenicol antibiotic	antibiotic efflux	99.76	100.00
Strict	emrA		protein homolog model	major facilitator superfamily (MFS) antibiotic efflux pump	fluoroquinolone antibiotic	antibiotic efflux	97.69	100.00
Strict	Escherichia coli ampH beta-lactamase		protein homolog model	ampC-type beta-lactamase	cephalosporin, penam	antibiotic inactivation	97.92	100.00
Strict	Escherichia coli EF-Tu mutants conferring resistance to Pulvomycin	R234F	protein variant model	elfamycin resistant EF-Tu	elfamycin antibiotic	antibiotic target alteration	99.49	96.33
Strict	Escherichia coli marR mutant conferring antibiotic	Y137H, G103S, S3N	protein overexpression model	resistance-nodulation-cell division (RND) antibiotic efflux pump	fluoroquinolone antibiotic, cephalosporin, glycylcycline, penam, tetracycline antibiotic, rifamycin	antibiotic target alteration, antibiotic efflux	94.44	100.00

	resistance				antibiotic, phenicol antibiotic, triclosan			
Strict	Escherichia coli mdfA		protein homolog model	major facilitator superfamily (MFS) antibiotic efflux pump	tetracycline antibiotic, benzalkonium chloride, rhodamine	antibiotic efflux	93.41	100.00
Strict	kdpE		protein homolog model	kdpDE	aminoglycoside antibiotic	antibiotic efflux	96.44	100.00
Strict	marA		protein homolog model	resistance-nodulation-cell division (RND) antibiotic efflux pump, General Bacterial Porin with reduced permeability to beta-lactams	fluoroquinolone antibiotic, monobactam, carbapenem, cephalosporin, glycylcycline, cephamycin, penam, tetracycline antibiotic, rifamycin antibiotic, phenicol antibiotic, triclosan, penem	antibiotic efflux, reduced permeability to antibiotic	95.28	100.00
Strict	mdtA		protein homolog model	resistance-nodulation-cell division (RND) antibiotic efflux pump	aminocoumarin antibiotic	antibiotic efflux	96.87	100.00

Strict	mdtB		protein homolog model	resistance-nodulation-cell division (RND) antibiotic efflux pump	aminocoumarin antibiotic	antibiotic efflux	98.94	100.00
Strict	mdtC		protein homolog model	resistance-nodulation-cell division (RND) antibiotic efflux pump	aminocoumarin antibiotic	antibiotic efflux	98.05	100.00
Strict	PmrF		protein homolog model	pmr phosphoethanolamine transferase	peptide antibiotic	antibiotic target alteration	97.5	100.00
Strict	baeR		protein homolog model	resistance-nodulation-cell division (RND) antibiotic efflux pump	monobactam,	antibiotic inactivation	98.74	105.83
Strict	kdpE		protein homolog model	kdpDE	cephalosporin	antibiotic inactivation	96.44	100.00
Strict	marA		protein homolog model	resistance-nodulation-cell division (RND) antibiotic efflux pump, General Bacterial Porin with reduced permeability to beta-lactams	antibiotic efflux, reduced permeability to antibiotic	antibiotic efflux	95.28	100.00
Strict	Escherichia coli EF-Tu mutant	R234F	protein variant model	elfamycin resistant EF-Tu	elfamycin antibiotic	antibiotic target alteration	99.49	96.33

	s conferring resistance to Pulvomycin							
Strict	Escherichia coli marR mutant conferring antibiotic resistance	Y137H, G103S, S3N	protein overexpression model	resistance-nodulation-cell division (RND) antibiotic efflux pump	fluoroquinolone antibiotic, cephalosporin, glycylcycline, penam, tetracycline antibiotic, rifamycin antibiotic, phenicol antibiotic, triclosan	antibiotic target alteration, antibiotic efflux	94.44	100.00
