# Supplementary material for: Pan-Genomics of Escherichia albertii for Antibiotic Resistance Profiling in Different Genome Fractions and Natural Product Mediated Intervention: In Silico Approach
Source: Life (Basel). 2023 Feb 15;13(2):541. doi: 10.3390/life13020541 (PMC9962377; doi:10.3390/life13020541)
Supplement: Supplementary file 1 [file life-13-00541-s001.zip › Supplementary Table S3.pdf]

Supplementary Table S3. Antibiotic resistance gene from the unique genome fraction.

Unique Resistome								
**RGI Criteria**	**ARO Term**	**SNP**	**Detection Criteria**	**AMR Gene Family**	**Drug Class**	**Resistance Mechanism**	**% Identity of Matching Region**	**% Length of Reference Sequence**
Perfect	APH(4)-Ia		protein homolog model	APH(4)	aminoglycoside antibiotic	antibiotic inactivation	100.0	100.00
Perfect	CTX-M-55		protein homolog model	CTX-M beta-lactamase	cephalosporin	antibiotic inactivation	100.0	100.00
Perfect	AAC(3)-IId		protein homolog model	AAC(3)	aminoglycoside antibiotic	antibiotic inactivation	100.0	100.00
Perfect	linG		protein homolog model	lincosamide nucleotidyltransferase (LNU)	lincosamide antibiotic	antibiotic inactivation	100.0	100.00
Perfect	APH(3')-IIa		protein homolog model	APH(3')	aminoglycoside antibiotic	antibiotic inactivation	100.0	100.00
Perfect	AAC(3)-IV		protein homolog model	AAC(3)	aminoglycoside antibiotic	antibiotic inactivation	100.0	100.00
Perfect	QnrS1		protein homolog model	quinolone resistance protein (qnr)	fluoroquinolone antibiotic	antibiotic target protection	100.0	100.00
Perfect	ANT(2")-Ia		protein homolog model	ANT(2")	aminoglycoside antibiotic	antibiotic inactivation	100.0	100.00

Perfect	dfrA12		protein homolog model	trimethoprim resistant dihydrofolate reductase dfr	diaminopyrimidine antibiotic	antibiotic target replacement	100.0	100.00
Strict	adeF		protein homolog model	resistance-nodulation-cell division (RND) antibiotic efflux pump	fluoroquinolone antibiotic, tetracycline antibiotic	antibiotic efflux	61.15	99.15
Strict	tetM		protein homolog model	tetracycline-resistant ribosomal protection protein	tetracycline antibiotic	antibiotic target protection	99.37	100.00
Strict	oqxA		protein homolog model	resistance-nodulation-cell division (RND) antibiotic efflux pump	fluoroquinolone antibiotic, glycylcycline, tetracycline antibiotic, diaminopyrimidine antibiotic, nitrofuran antibiotic	antibiotic efflux	99.74	100.00
Strict	AAC(3)-VIa		protein homolog model	AAC(3)	aminoglycoside antibiotic	antibiotic inactivation	99.67	100.00
